# Complete mitochondrial genome of *Qiongphasma jianfengense obtusicristata* (Phasmatodea: Lonchodidae: Necroscinae)

**DOI:** 10.1128/mra.00615-25

**Published:** 2025-08-12

**Authors:** Jing Guo, Ting Luo, Xun Bian

**Affiliations:** 1 Key Laboratory of Ecology of Rare and Endangered Species and Environmental Protection (Guangxi Normal University), Ministry of Education, Guilin, China; Rochester Institute of Technology, Rochester, New York, USA

**Keywords:** mitogenome, China, Necroscinae, *Qiongphasma*

## Abstract

Here, the complete mitochondrial genome of *Qiongphasma jianfengense obtusicristata* was sequenced and annotated from China. The circular genome has a size of 16,747 bp and contains 13 protein-coding genes, 22 tRNA genes, and 2 rRNA genes. The complete mitochondrial genome of *Q. jianfengense obtusicristatai* provides basic genome data for relative studies.

## ANNOUNCEMENT

As the southernmost island of China, Hainan Island’s isolated environment restricts gene flow (1). This drives the evolution of unique traits in its stick insects, resulting in endemic *Qiongphasma* species (2, 3). As a phytophagous species, the population dynamics of *Q. jianfengense obtusicristata* Ho, 2013, serves as a bioindicator of the health and stability of the local forest ecosystem (4, 5). Currently, there are 66 complete mitochondrial genomes available for Phasmatodea (6–10). We present one complete mitogenome of the genus *Qiongphasma*.

The male specimen of *Q. jianfengense obtusicristata* analyzed in this paper was collected from Jianfengling, Hainan, China (18.731°N 108.872°E) and preserved in anhydrous alcohol at −4°C in Guangxi Normal University. Total genomic DNA was extracted from the muscle tissue of postfemora using the genomic DNA extraction kit (TIANGEN Biochemical Technology) and sequenced using the DNBSEQ next-generation sequencing platform (Shenzhen BGI Genomics Co., Ltd.) with PE150 (paired-end 150 bp) sequencing strategy. Using SOAPnuke software (11), reads with lengths <150 bp were removed, those containing ≥0.1% N bases were eliminated, and reads with polyA/T/G/C stretches >50 bp were excluded, yielding 20,018,923 clean reads. The complete mitogenome sequence was assembled by NOVOPlasty 4.2.1 software (12) and *Neohirasea hongkongensis* (NC_087839.1) (13) was selected as a reference sequence. The assembled mitogenome was annotated on the MITOS webserver (14) and using MEGA v.11 (15) to examine the 13 protein-coding genes (PCGs) loci. The 13 PCGs could be correctly translated into proteins via MEGA v.11 (15). AT content, AT skew, and GC skew were calculated using PhyloSuite v1.2.3 (16). A circular mitogenome map was generated using the Chloroplot server online tool (17).

**Fig 1:**
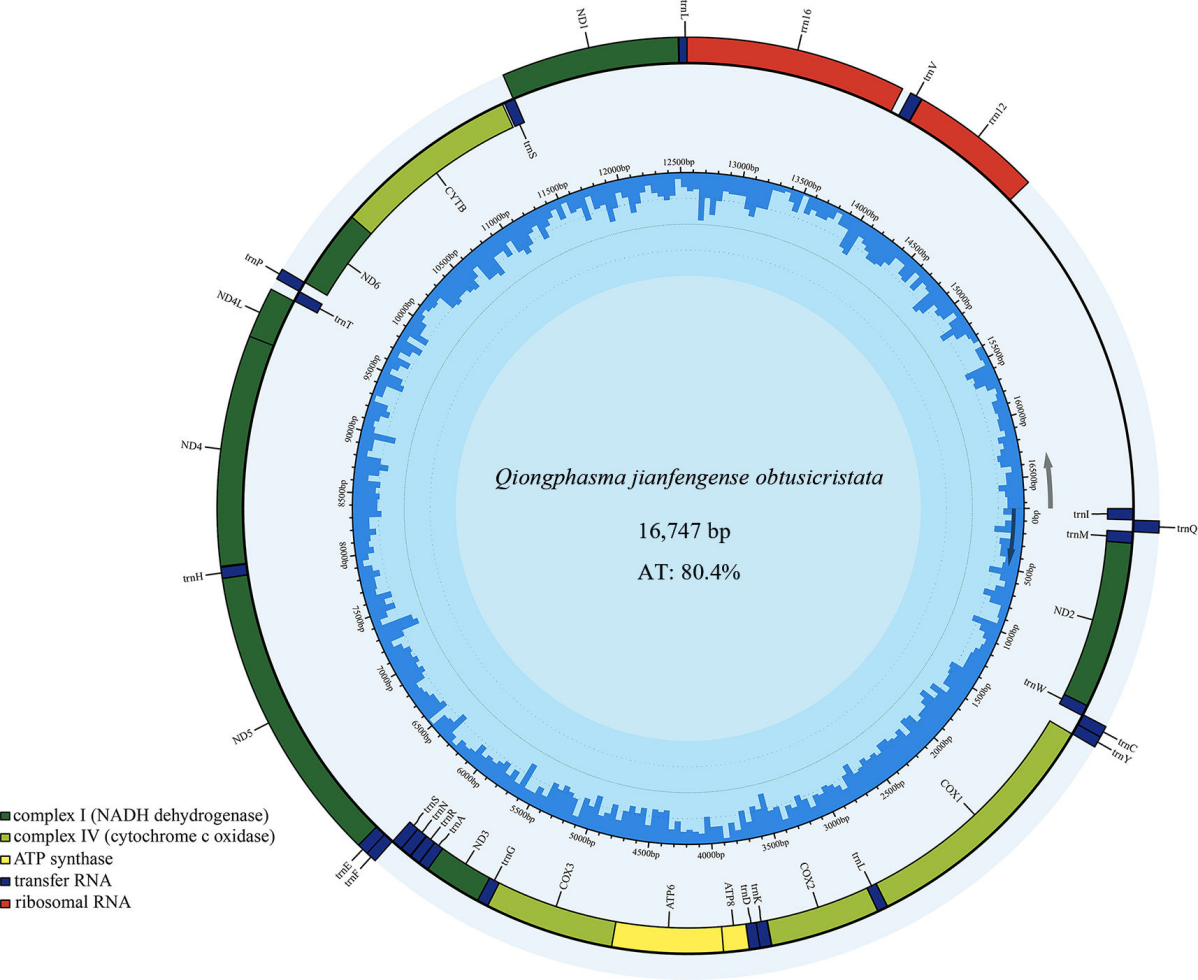
Map of the mitochondrial genome of *Q. jianfengense obtusicristata*. Overlapping features are excluded for a better graphical representation of the architecture of the genome. Genes on the outside are transcribed counterclockwise, and genes on the inside are transcribed clockwise as informed by the arrows in the figure. The different colors in the outermost ring correspond to different genes.

The complete mitogenome (GenBank accession number PV630663) of *Q. jianfengense obtusicristata* was 16,747  bp in length and contains 13 PCGs, 22 tRNAs, 2 rRNAs, and a control region (CR) (Fig. 1). The mitogenome is AT-rich (80.4%). All the 13 PCGs have a typical ATN start codon and are terminated with complete TAA, except NAD2, COX1, COX3, NAD3, and NAD5, which end with incomplete TA or T. The transcription of TA or T may involve poly(A) tail addition to compensate for the deficiency and ensure translation termination (18). The tRNAs ranged in length from 63 bp (tRNA-Pro) to 72 bp (tRNA-Leu). The lengths of 16S rRNA and 12S rRNA in *Q. jianfengense obtusicristata* are 1,267 bp and 766 bp (Table 1). The whole genome (0.124), J-strand tRNAs (0.08), tRNA genes (0.022), and J-strand PCGs (0.017) are AT-skewed. The GC skew of PCGs (0.018), N-strand PCGs (0.231), tRNA genes (0.157), J-strand tRNAs (0.043), N-strand tRNAs (0.327), and rRNAs (0.303) is positive. The comparative analysis revealed that the whole-genome positive AT skews (0.124) and negative GC skews (−0.164) were observed.

**TABLE 1:** Organization of the mitochondrial genome of *Q. jianfengense obtusicristata*

Gene	Type	Start	Stop	Size (bp)	Start codon	Stop codon	Direction
tRNA-Ile	tRNA	1	68	68	–^*a*^	–	Forward
tRNA-Gln	tRNA	71	139	69	–	–	Reverse
tRNA-Met	tRNA	140	208	69	–	–	Forward
NAD2	CDS	209	1,223	1,015	ATT	T	Forward
tRNA-Trp	tRNA	1,224	1,290	67	–	–	Forward
tRNA-Cys	tRNA	1,283	1,349	67	–	–	Reverse
tRNA-Tyr	tRNA	1,350	1,414	65	–	–	Reverse
COX1	CDS	1,416	2,949	1,534	ATG	T	Forward
tRNA-Leu	tRNA	2,950	3,013	64	–	–	Forward
COX2	CDS	3,014	3,682	669	ATA	TAA	Forward
tRNA-Lys	tRNA	3,685	3,758	74	–	–	Forward
tRNA-Asp	tRNA	3,754	3,819	66	–	–	Forward
ATP8	CDS	3,820	3,978	159	ATT	TAA	Forward
ATP6	CDS	3,975	4,649	675	ATG	TAA	Forward
COX3	CDS	4,649	5,433	785	ATG	TA	Forward
tRNA-Gly	tRNA	5,434	5,501	68	–	–	Forward
NAD3	CDS	5,502	5,853	352	ATA	T	Forward
tRNA-Arg	tRNA	5,854	5,917	64	–	–	Forward
tRNA-Ala	tRNA	5,919	5,982	64	–	–	Forward
tRNA-Asn	tRNA	5,987	6,054	68	–	–	Forward
tRNA-Ser	tRNA	6,055	6,122	68	–	–	Forward
tRNA-Phe	tRNA	6,124	6,187	64	–	–	Forward
tRNA-Glu	tRNA	6,186	6,252	67	–	–	Reverse
NAD5	CDS	6,253	7,975	1,723	ATT	T	Reverse
tRNA-His	tRNA	7,976	8,039	64	–	–	Reverse
NAD4	CDS	8,045	9,376	1,332	ATG	TAA	Reverse
NAD4L	CDS	9,370	9,663	294	ATG	TAA	Reverse
tRNA-Thr	tRNA	9,666	9,729	64	–	–	Forward
tRNA-Pro	tRNA	9,730	9,792	63	–	–	Reverse
NAD6	CDS	9,794	10,276	483	ATC	TAA	Forward
CYTB	CDS	10,276	11,412	1,137	ATG	TAA	Forward
tRNA-Ser	tRNA	11,425	11,490	66	–	–	Forward
NAD1	CDS	11,484	12,503	1,020	ATA	TAA	Reverse
tRNA-Leu	tRNA	12,507	12,578	72	–	–	Reverse
16S rRNA	tRNA	12,553	13,819	1,267	–	–	Reverse
tRNA-Val	tRNA	13,871	13,941	71	–	–	Reverse
12S rRNA	tRNA	13,945	14,710	766	–	–	Reverse
CR	–	14,711	16,747	–	–	–	–

^*a*^ –, not applicable.

## Data Availability

The complete mitochondrial genome sequence of *Qiongphasma jianfengense obtusicristata* is available in GenBank under accession number PV630663. The associated BioProject, SRA, and BioSample numbers are PRJNA1262732, SRR33561847, and SAMN48480518, respectively. The mitochondrial genome referenced in the text is *Neohirasea hongkongensis*. GenBank accession number NC 087839.1.
